# Supplementary material for: Asynchronous magnetic resonance elastography: Shear wave speed reconstruction using noise correlation of incoherent waves
Source: Magn Reson Med. 2022 Oct 27;89(3):990–1001. doi: 10.1002/mrm.29502 (PMC9792433; doi:10.1002/mrm.29502)
Supplement: Supplementary file 1 — DATA S1 MATLAB code used to generate the numerical simulations of Figure 1 [file MRM-89-990-s002.zip › k-Wave/helpfiles/scaleTime.html]

scaleTime :: Functions (k-Wave)


# scaleTime

Convert seconds to hours, minutes, and seconds.

## Syntax

```
time = scaleTime(seconds)
```

## Description

`scaleTime` converts an integer number of seconds into hours, minutes, and seconds, and returns a string with this information.

## Inputs

|  |  |
| --- | --- |
| `seconds` | number of seconds |

## Outputs

|  |  |
| --- | --- |
| `x_sc` | string of scaled time |

## See Also

`scaleSI`
